# The Patterns of Drivers' Traffic Behavior: Evidence From Three Countries

**DOI:** 10.3389/fpsyg.2022.869029

**Published:** 2022-04-07

**Authors:** Tatiana V. Kochetova

**Affiliations:** Faculty of Social Psychology, Moscow State University of Psychology and Education, Moscow, Russia

**Keywords:** pattern of behavior, risky traffic behavior, driving, road traffic environment, person-environment interaction (P-E)

## Introduction

Numerous studies of driver behavior in the road traffic environment demonstrates a high interest in the problem of an individual behavior in a high-risk environment and a significant increase in the number of works in this area. This increase is also due to official statistics of accidents in the traffic environment. The methodological rationale for such work is related to an interdisciplinary approach that allows us to consider various aspects of the behavior of road users in the conditions of road traffic environment.

Summarizing these works, we can distinguish at least two aspects that characterize them: 1) research of risky traffic behavior and its predictors—individual psychological determinants (Šucha and Cernochová, 2016; Suhr and Dula, 2017; Lemarié et al., 2019; Songa et al., 2021); 2) research of the specific features of traffic climate—social psychological determinants (Omidi et al., 2021), which lay ground for risky traffic behavior leading to traffic accident.

Along with this, it is worth highlighting separate works devoted to the study of “models of driving behavior” (Ranney, 1994), as well as “driving patterns” or “social interaction patterns in driver behavior” (Wilde, 1976) characterized by a high level of risk. It is possible that the study of behavior patterns in this context largely goes through the tradition laid down in the works of K. Lewin, who considered aggressive behavior as a social behavior pattern as an example (Lewin et al., 1939). Thus, Lewin proposed to consider any behavior as a function of interaction between the person and the environment, while patterns as a unit of such interaction per unit of time (Lewin, 1935).

Therefore, it seems logical that the study of models of risky traffic behavior of drivers should focus on the study of the *content, structural*, and *dynamic* aspects of behavior. The first implies consideration of the “boundaries” of risky driver behavior and its difference from, for example, aggressive or dangerous driving (this aspect can be called *topological*—author's note). The second provides a basis for studying the relationships between various behavioral manifestations of patterns in a broad social context and allows identifying risky road behavior as such (Householder, 1939). And finally, the third defines the stability and dynamics of behavior change in future.

## Development of Research Methodology

In recent years, the authors of some psychological studies in the field of a psychologically safe environment began to interpret behavior as a result of person-environment interaction (P-E). The authors emphasize that the theoretical concepts of person-environment interaction open up great opportunities for studying not risky, but safe human behavior in an anthropogenic, artificially created environment (Naweed et al., 2020). Using the organizational environment as an example, they explain that “the generation of personal safety behaviors is not only related to characteristics of an individual, but is also closely related to his/her environment. If the environment is consistent with individuals' behavior, it will often contribute to the development of such behavior” (Wang et al., 2021). Thus, Wang et al. (2021) attribute the practical significance of such research to the formation of a congruent safe environment and, thereby, reducing behavioral risks.

Considering that the road transport environment is an anthropogenic environment created by man, it is quite logical that the interaction of a person (driver) with such a high-risk environment can be considered as a special case of person-environment interaction (Naweed et al., 2020). In this regard, the existing theoretical ideas about the models and patterns of risky traffic behavior of drivers can be expanded and supplemented on the basis of the theory of person-environment interaction.

## Design and Methods

Based on the theoretical ideas formulated by Lewin that behavior is a function of the interaction between the individual and the environment (Lewin, 1935), as well as on the basic ideas of the theory of person-environment interaction, one can set a certain framework (“research vector”) for considering patterns of risky traffic behavior of drivers (Naweed et al., 2020).

After a brief review of the key important developments in the study of high-risk behavior as a result of human interaction with the environment, we move on toward a conceptual understanding of patterns of traffic behavior of drivers with an emphasis on the process of interaction of the driver with the traffic environment.

Thus, the risky traffic behavior can be identified by the leading role of a particular behavior pattern and its connection with some other patterns (Wilde, 1976; Ranney, 1994). Some researchers draw attention to the relationship between these behavioral patterns and the propensity to drink alcohol (Alcañiza et al., 2016). At the same time risky traffic behavior can be observed in a combination of various behavioral patterns without alcohol consumption (Lajunen et al., 2004). However, it is the use of alcohol that can significantly increase accident risks, consequently such traffic behavior can be considered more risky than any other behavior pattern (Kochetova and Meinhard, 2020). Research of a driver's traffic behavior can provide a comprehensive picture of the peculiarities of his/her movement in a certain traffic environment and interaction with other road users. Risky traffic behavior includes participation in illegal high-speed competitions (street racing), driving without driving license or ignoring traffic police signals, non-compliance with traffic legislation, and alcohol abuse in various social situations, indirectly linked to driving (Meinhard, 2019). In other words, there may be some comorbidity of various behavioral features that define drivers' traffic behavior as “high-risk,” deviating from the traffic laws and the civil legal norms (Meinhard, 2019, 2020). It is a combination of individual actions—steady behavioral patterns in the road traffic—with other features of drivers' behavior outside the context of driving performance that defines his or her traffic behavior as a whole.

These ideas are reflected in the “Traffic Risk Evaluation Model” by Meinhard (2019), where the Model is focused on structural and content aspects of risky traffic behavior of drivers (The dynamic of risky behavioral patterns might study through test retest method in research). The Model has been under implementation in Traffic Offenders Prevention Program in Estonia since 2014. The Model allows to find correlations between different behavioral patterns of risky traffic behavior, which make it possible to characterize this behavior holistically. This Model includes two components: (I) the questionnaire containing six main scales that measure behavioral patterns, which are interconnected with (II) the AUDIT scale used in world practice, i.e., the propensity to drink alcohol (Babor et al., 2010). The scales of the questionnaire are the following: (1) “Attitude to drunk driving,” (2) “Threats and risks of driving,” “Non-alcohol offenses,” (3) “Risks as a driver,” (4) “Risks as a passenger,” (5) “Classical offenses in the road traffic,” and (6) “Misconduct outside road traffic” for evaluation behavior outside road traffic). One can see that five of these scales characterize the personal-environmental interaction. Thus, scales 3 and 4 characterize the interaction of an individual with his/her environment (driver-passenger, passenger-driver), while scales 2 and 5 characterize the interaction of an individual with a road transport environment. Scale 6 characterizes the interaction of an individual with a wider social environment (legislation, formal rules, and social-cultural norms)^1^.

Using this Model, we decided to conduct a pilot study and consider the characteristics of risky traffic behavior of drivers in three different countries: Estonia, Russia, and Kazakhstan. It should be emphasized that the choice of these countries was not random, but justified by a number of factors: (1) all three are Post-Soviet states, which determines a certain cultural commonality and similarity of cultural values; (2) each of these countries over the past 30 years has gone through its own “exclusively” individual path of development, which could not but affect the social-cultural norms of the environment of each country.

The first attempt to validate the questionnaire on a Russian sample was made as part of the pilot project “Approbation of the method of psychological screening of drivers prone to risky traffic behavior” (Kochetova and Meinhard, 2020). However, a more detailed study of the behavioral patterns of risky traffic behavior of drivers using the “Traffic Risk Evaluation Model” and the questionnaire was not carried out. For cross-cultural validation, a study of samples from three different countries was initiated. In this pilot study for all three samples the Russian language version of the questionnaire was offered to Russian speaking citizens in Estonia, Russia, and Kazakhstan. This study design was based on the works devoted to validation of another widely used driver's behavior questionnaire “The Manchester Driver Behavior Questionnaire” (DBQ) which allows to measure various aggressive violations of drivers, errors of driving, etc. (Lajunen et al., 2004; Martinussen et al., 2013; Bener et al., 2016). Based on the results presented in these works, it can be stated that, for example, to analyze the factor structure of DBQ, samples of at least three different countries were used (Lajunen et al., 2004).

The aim of this paper is to outline the framework for further studies that would allow to make conclusions about differences in traffic behavior of drivers, including evidence about the equivalence of the structure and items of the scale in various countries and to compare the average values of the latent factors. So far in this study, the total sample includes three groups from different countries: Estonia (*n* = 4,061), Russia (*n* = 453), and Kazakhstan (*n* = 79). There are plans to expand the Kazakh sample to ensure representativeness.

According to the methodology of the person-environment interaction, the social risk indicators may be reflected in the official road accident statistics and, it would be logical to suggest that interaction of road users with transport environment would be different in different countries.

Thus, even the preliminary data in risky traffic behavior studies allows to assume that the interaction of an individual with the social-cultural environment can determine some characteristics of behavior (Nordfjærn et al., 2011). This assumption, in our opinion, can serve as a basis for planning a more detailed and scrupulous study of the patterns of risky traffic behavior in various socio-cultural environments.

## Discussion and Implications

The above mentioned studies of personal-environmental interaction (P-E) in an anthropogenic, artificially created environment can make a basis for further replication of models for assessing the risky behavior of an individual and a detailed study of the patterns that characterize his/her behavior in road environment. The aim of this work was not to present the results of the original research but to put forward the idea of the person-environment interaction approach in future studies of risky traffic behavior.

The conceptual framework presented here advances previous theoretical studies of risky traffic behavior of drivers as an option of person-environment interaction in a road traffic environment and opens up broad perspectives for studying patterns of behavioral risks. A meaningful analysis of individual aspects of this process can include driving patterns, social interaction patterns in driver behavior, patterns of interaction with the social environment while driving (traffic patterns as a passenger) (Simons-Morton et al., 2011), patterns of offenses in the road traffic environment, and patterns of offenses outside the context of traffic—social surroundings. The “Traffic Risk Evaluation Model” is presented as a possible research Model aimed at studying various combinations of driver's behavioral patterns in the road traffic environment—a particular case of risky person-environment interaction. Despite current limitations, the original samples might be used for quality evaluation of the questionnaire and further development of the Model in various countries. It appears that the prospects for cross-cultural validation of the Model developed by Meinhard should include the following: (1) description of the psychometric properties; (2) internal consistency of the scale scores including calculation of Cronbach's alpha reliability coefficients; (3) analysis of the equivalence of questionnaire factor structures in three countries and comparison of these factors. When necessary, the items and scales should be adapted to socio-cultural norms (legislation, formal rules, and social norms), including the adaptation of the “Standard drink” items of the AUDIT scale, to the metric system of each country.

## Author Contributions

TK: conceptualization, investigation, methodology, and writing – original draft.

## Conflict of Interest

The author declares that the research was conducted in the absence of any commercial or financial relationships that could be construed as a potential conflict of interest.

## Publisher's Note

All claims expressed in this article are solely those of the authors and do not necessarily represent those of their affiliated organizations, or those of the publisher, the editors and the reviewers. Any product that may be evaluated in this article, or claim that may be made by its manufacturer, is not guaranteed or endorsed by the publisher.

## Footnotes

^1^All rights to use this questionnaire are reserved by G. Meinhard, TMÜ “Trafity OÜ” (Tartu; Estonia).
